# Code Blue Awareness in Dental Students: A Pilot Project to Develop Awareness in the Clinic

**DOI:** 10.1111/eje.70133

**Published:** 2026-03-15

**Authors:** Fatih Oluş, Hüseyin Babun

**Affiliations:** ^1^ Department of Oral and Maxillofacial Surgery, Faculty of Dentistry Akdeniz University Antalya Turkiye

**Keywords:** cardiopulmonary resuscitation, clinical training, Code Blue, dental education, emergency response

## Abstract

**Introduction:**

The “Code Blue” system is a standardised emergency response protocol designed to ensure rapid intervention in critical medical situations, particularly cardiopulmonary arrest. Although mandatory in Türkiye since 2011, inappropriate activations still occur, often due to limited awareness among healthcare staff, including dental students. This pilot project aimed to assess dental students' knowledge and awareness regarding Code Blue and to examine whether these outcomes differ according to the level of clinical training.

**Materials and Methods:**

A cross‐sectional survey was conducted among 186 dental students at Akdeniz University, including 3rd‐year (preclinical) and 5th‐year (clinical) students. A structured 10‐item multiple‐choice questionnaire was used to evaluate participants' knowledge of Code Blue indications, activation procedures, and response expectations. Descriptive and comparative statistical analyses were performed.

**Results:**

Of the 186 students analysed, 53.2% were female and 46.8% were male. The most recognised emergency code was Code White (82.26%), followed by Code Blue (70.43%). Code Blue awareness was significantly higher among fifth‐year students (77.3%) than third‐year students (62.9%) (*p* < 0.05). Fifth‐year students also demonstrated greater knowledge of Code Blue indications and were less likely to choose “don't know.” However, common misinterpretations were observed: 70.49% incorrectly identified vasovagal syncope as a Code Blue condition, and only 7.38% correctly rejected all non‐indicated scenarios. Knowledge of basic life support (BLS) content did not significantly differ between groups (*p* = 0.8153).

**Conclusions:**

Code Blue knowledge and awareness were higher among students with greater clinical exposure; however, overall awareness remained limited. These findings support earlier and more structured integration of institutional emergency code training, simulation‐based reinforcement, and practical emergency preparedness training within the dental curriculum. Further multi‐center studies are recommended to enhance generalizability and guide educational interventions.

## 1 Introduction

The “Code Blue” system is a hospital‐based emergency response protocol designed to ensure rapid intervention in life‐threatening medical events, particularly cardiopulmonary arrest. It facilitates immediate action by mobilising a team of trained healthcare professionals to assist patients, relatives, or hospital personnel as efficiently as possible [1]. Among various colour‐coded emergency responses, Code Blue stands out as the only universally recognised code for the same clinical scenario across the globe.

A Code Blue team typically comprises physicians and nurses who are specially trained in cardiopulmonary resuscitation (CPR) and advanced life support techniques. This system was first implemented in the United States and later adopted in many countries as a standard component of in‐hospital emergency protocols [2]. In Türkiye, the implementation of Code Blue was institutionalised in 2011 with the publication of the “Patient and Employee Safety Regulation,” which made it mandatory for all healthcare facilities [3].

Cardiac arrest is a critical event that frequently occurs in hospital settings, and delays in intervention are strongly associated with increased mortality and poor neurological outcomes [4, 5]. As a response, dedicated Code Blue teams have been established in hospitals across the country to minimise preventable in‐hospital deaths.

Although healthcare institutions provide training on emergency codes, inappropriate Code Blue activations are still common. These are often due to a lack of awareness or misunderstanding of the system's purpose, activation criteria, and scope. Dental faculties, in particular, may pose a unique challenge due to the limited exposure of students to acute medical emergencies, especially during preclinical years.

The primary objective of this study is to evaluate the knowledge and awareness levels of dental students regarding the Code Blue emergency response system. Specifically, the study aims to assess their readiness to correctly identify and respond to situations requiring Code Blue activation. By comparing students at different stages of their education—namely, preclinical (3rd‐year) and clinical (5th‐year) students—this study seeks to determine whether clinical exposure enhances preparedness. Additionally, the findings will help identify potential gaps in emergency training within the dental curriculum and contribute to the development of targeted educational strategies to improve emergency protocol implementation and awareness among future dental professionals.

## 2 Materials and Methods

This cross‐sectional descriptive study was conducted at the Faculty of Dentistry, Akdeniz University, during the 2022–2023 academic year. The study population consisted of 3rd‐ and 5th‐year dental students. While 3rd‐year students represented the preclinical group with no clinical exposure, 5th‐year students were considered the clinical group due to their active participation in patient care.

### 2.1 Questionnaire Development and Psychometrics

A 10‐item knowledge test was developed from the institution's Code Blue protocol and Basic Life Support (BLS) materials, aligned with current national guidance on in‐hospital emergency response. Content validity was assessed by a five‐member expert panel using a 4‐point relevance scale; item‐level CVI (I‐CVI), scale‐level CVI/Average (S‐CVI/Ave), and modified kappa were calculated, and inadequate items were revised. Brief cognitive interviews (3rd‐year *n* = 23; 5th‐year *n* = 27) confirmed comprehensibility. Items covered five domains: Code Blue definition, activation criteria and call number, frequently misinterpreted non‐indications, and immediate role/BLS actions (AED use, CAB sequence).

At the time of the study, Code Blue protocols were not delivered as a structured, stand‐alone didactic course within the preclinical curriculum. BLS principles were covered within broader theoretical sessions; however, specific institutional emergency code activation procedures were not systematically reinforced during the third year. Exposure to Code Blue procedures primarily occurred during clinical rotations in the later years of training.

The survey was administered both electronically and via printed forms to accommodate student accessibility. Participation was voluntary, and informed consent was obtained from all respondents. Responses were anonymized and subsequently digitized for statistical evaluation.

Data analysis was performed using IBM SPSS Version 25 software. Descriptive statistics (frequencies, percentages) were used to summarise the results. Comparative analyses between 3rd‐ and 5th‐year students were conducted using chi‐square tests for categorical variables. A *p* < 0.05 was considered statistically significant.

Ethics Statement for this study was obtained from the Clinical Research Ethics Committee of Akdeniz University [TBAEK—280].

## 3 Results

In this study, data from a total of 186 students who completed the questionnaire in full were analysed. Regarding gender distribution, female students (53.2%) slightly outnumbered male students (46.8%). When examined by academic year, 52.8% of third‐year students were female and 47.2% were male, while among fifth‐year students, 54.6% were female and 45.4% were male (Table 1).

**TABLE 1:** Distribution of students by academic year and ender.

Academic year	Female		Male	
	*n*	%	*n*	%
3	47	52.8	42	47.2
5	53	54.6	44	45.4
Total	100	53.2	86	46.8

*Note:* %: Column percentage.

Among the students, the most recognised emergency code was the Code White, which refers to violence against healthcare workers; it was recognised by 153 students, corresponding to a recognition rate of 82.26%. The Code Blue, used in cases of cardiopulmonary arrest, was identified by 131 students (70.43%). The Code Red, indicating a fire emergency, was identified by 96 students (51.61%), while the Code Pink, referring to infant or child abduction, was known by 50 students (26.88%). Less commonly recognised were the Code Orange, signifying chemical hazards, recognised by only 15 students (8.06%), the Code Black, indicating a bomb threat, recognised by 13 students (6.99%), and the Code Grey, used for armed assailants or hostage situations, identified by only 6 students (3.23%) (Table 2).

**TABLE 2:** Recognition rates of emergency codes.

Code colour	Aware		Unaware	
	*n*	%	*n*	%
Blue	131	70.43	55	29.57
White	153	82.26	33	17.74
Red	96	51.61	90	48.39
Pink	50	26.88	136	73.12
Orange	15	8.06	171	91.94
Black	13	6.99	173	93.01
Grey	6	3.23	180	96.77

*Note:* %: Column percentage.

Specifically focusing on Code Blue awareness, only 62.9% of third‐year students reported being aware of the Code Blue, compared to 77.3% of fifth‐year students. This difference between academic years was statistically significant (*p* < 0.05) (Table 3).

**TABLE 3:** Code Blue awareness rates by academic year.

Academic year	Code Blue				Test statistic	*p*
	Aware		Unaware			
	*n*	%	*n*	%		
3	56	62.9	33	37.1	5.337	0.0209*
5	75	77.3	22	22.7		

* *p* < 0.05 indicates statistical significance. %: Column percentage.

When Code Blue indication knowledge was evaluated by academic year, 57.3% of third‐year students responded correctly, 5.62% incorrectly, and 37.08% stated that they did not know. Among fifth‐year students, the distribution was 70.1% correct, 8.25% incorrect, and 21.65% “don't know.” These findings indicate that fifth‐year students have a higher level of knowledge regarding Code Blue indications compared to third‐year students, and the proportion of “don't know” responses is markedly lower among them (Table 4).

**TABLE 4:** Code Blue activation indication knowledge by academic year.

Academic year	Code Blue activation indication knowledge						Test statistic	*p*
	Correct		Incorrect		Don't know			
	*n*	%	*n*	%	*n*	%		
3	51	57.3	5	5.62	33	37.08	5.454	0.0654
5	68	70.1	8	8.25	21	21.65		

*Note:* %: Column percentage.

Among the students who responded to the scenarios requiring Code Blue activation, 70.49% identified vasovagal syncope as a condition warranting a Code Blue, while 29.51% did not consider it within the scope of Code Blue activation. Within the same sample, 49.18% evaluated hypotension/hypertension, 81.15% altered consciousness, 71.31% epileptic seizure, 19.67% agitation, and 48.36% falling as indications for a Code Blue. Notably, only 9 students (7.38% of the relevant respondents) correctly answered “No” to all six scenarios, demonstrating an accurate understanding of appropriate Code Blue indications (Table 5).

**TABLE 5:** Student evaluation of incorrect Code Blue activation scenarios.

Clinical scenarios	Code Blue activation			
	Yes		No	
	*n*	%	*n*	%
Vasovagal syncope	86	70.49	36	29.51
Altered consciousness	99	81.15	23	18.85
Epileptic seizure	87	71.31	35	28.69
Hypotension/hypertension	60	49.18	62	50.82
Fall	59	48.36	63	51.64
Agitation	24	19.67	98	80.33

*Note:* %: Column percentage. Denominators vary due to item‐level nonresponse.

When responses to the question “What does basic life support consist of?” were analysed by academic year, 42.7% of third‐year students answered correctly, 47.19% incorrectly, and 10.11% selected “don't know.” Among fifth‐year students, 38.15% responded correctly, 50.51% incorrectly, and 11.34% selected “don't know.” According to the chi‐square test, there was no statistically significant difference between the two groups (*p* = 0.8153) (Table 6).

**TABLE 6:** Student responses to the content of basic life support by academic year.

Academic year	Content of Basic Life Support (BLS)						Test statistic	*p*
	Correct		Incorrect		Don't know			
	*n*	%	*n*	%	*n*	%		
3	38	42.7	42	47.19	9	10.11	0.408	0.8153
5	37	38.15	49	50.51	11	11.34		

*Note:* %: Column percentage.

Among third‐year students, 47.19% reported being unaware of the “Code Blue activation phone number”, compared to 25.77% of fifth‐year students. The proportion of students who selected the incorrect number, “1122,” was 12.36% among third‐year students and 11.34% among fifth‐year students. The correct activation number, “2222,” was identified by 22.47% of third‐year students and 44.33% of fifth‐year students. The option “3333” was selected by 4.49% of third‐year students and 6.19% of fifth‐year students, while “4444” was chosen by 2.25% and 3.09%, respectively. Furthermore, 11.24% of third‐year students and 9.28% of fifth‐year students selected “9111.” A statistical comparison of the responses “I don't know” and the correct activation number “2222” between the two academic years revealed a significant difference (*p* < 0.05) (Table 7).

**TABLE 7:** Code Blue activation phone number knowledge by academic year.

Academic year	Code Blue activation phone number						Test statistic	*p*
	Correct (2222)		Incorrect		Don't know			
	*n*	%	*n*	%	*n*	%		
3	20	22.47	27	30.34	42	47.19	13.043	0.023*
5	43	44.33	29	29.9	25	25.77		

* *p* < 0.05 indicates statistical significance. %: Column percentage.

In response to the question regarding the responsibilities of the attending dentist during a Code Blue situation, 54.8% of third‐year students provided the correct answer, while this rate increased to 69.4% among fifth‐year students, indicating that fifth‐year students demonstrated a higher level of accuracy in identifying the dentist's responsibilities in such emergencies. Among respondents to these items, 82.5% indicated that chest compressions should be initiated until the emergency team arrives, while 17.5% disagreed. An overwhelming majority (98.33%) stated that relevant information should be communicated to the team upon their arrival. In contrast, opinions were more divided regarding assisting the team, with 57.5% responding affirmatively and 42.5% negatively. Lastly, only 24.17% agreed that the patient should be transferred to the intensive care unit after the dentist's intervention, whereas 75.83% disagreed with this statement (Table 8).

**TABLE 8:** Students' agreement on actions following Code Blue activation.

Actions following Code Blue activation	Yes		No	
	*n*	%	*n*	%
CPR Before team arrival	99	82.5	21	17.5
Informing the team upon arrival	118	98.33	2	1.67
Assisting the team during intervention	69	57.5	51	42.5
Transfer to ICU after intervention	29	24.17	91	75.83

*Note:* %: Column percentage. Denominators vary due to item‐level nonresponse.

## 4 Discussion

This study aimed to assess the knowledge and awareness levels of dental students regarding emergency code systems, particularly Code Blue, and their preparedness in responding to clinical emergencies. Overall, the results highlight several important findings that have significant implications for dental education and clinical training.

Firstly, awareness and knowledge of emergency codes varied significantly among the dental students surveyed. The most widely recognised code was the White Code, relating to incidents of violence against healthcare personnel. The high recognition rate of the White Code likely reflects the growing incidence of violence against healthcare professionals in our country, posing a genuine dilemma as to whether this heightened awareness, surpassing even the recognition of Code Blue, should be considered reassuring or concerning. Code Blue, which pertains specifically to cardiopulmonary arrest situations, was the second most recognised emergency code. However, recognition rates for other critical codes such as Orange (chemical hazards), Black (bomb threats), and Grey (armed assailant or hostage situations) were notably low. These findings align with previous literature, indicating healthcare students often have limited exposure and inadequate understanding of emergency response protocols, especially for scenarios encountered less frequently [6]. Given the critical nature of these codes for ensuring patient and healthcare provider safety, integrating comprehensive emergency code training into dental curricula is strongly recommended.

When examining knowledge differences by academic year, a statistically significant improvement in Code Blue awareness was observed among fifth‐year students compared to third‐year students. This progression likely reflects the cumulative effect of increased clinical exposure and practical training received in later stages of dental education. Similar trends have been reported in international studies, highlighting the importance of clinical experience and repeated practice in improving students' knowledge and response capabilities in emergencies [7]. Thus, the value of practical clinical training and repeated reinforcement of emergency response skills in dental curricula cannot be overstated.

The observed difference between third‐ and fifth‐year students may be partially explained by the structure of the current curriculum. While general BLS concepts are introduced during preclinical education, institutional emergency code protocols are not delivered as structured, stand‐alone learning objectives during the third year. As a result, awareness of Code Blue activation procedures appears to rely predominantly on incidental exposure during clinical rotations. These findings suggest that experiential learning alone may be insufficient and that earlier, structured integration of institutional emergency code training into the formal didactic curriculum may be necessary to ensure consistent preparedness among dental students.

Despite the improved awareness among senior students, substantial gaps remained regarding the appropriate indications for Code Blue activation. A considerable number of students incorrectly identified clinical situations such as vasovagal syncope, hypotension/hypertension, altered consciousness, epileptic seizures, agitation, and falling as warranting Code Blue activation. These misinterpretations point to fundamental misunderstandings regarding the precise scope of the Code Blue system, which should strictly be limited to cardiopulmonary arrest. This aligns with previous studies highlighting confusion among dental professionals regarding emergency activation criteria, potentially leading to inappropriate activations and inefficient resource use [8]. Targeted scenario‐based training sessions and simulation exercises have been recommended to address and correct these misunderstandings effectively.

Another significant finding was the very low proportion (7.38%) of students who correctly identified all presented scenarios as inappropriate for Code Blue activation. This reflects a broader issue related to inadequate comprehension and application of emergency protocols, underscoring the necessity of reinforcing critical decision‐making skills and clear differentiation between levels of medical emergencies in dental education programs. Previous studies similarly emphasize that dental professionals frequently face challenges in decision‐making during emergencies due to insufficient training and infrequent reinforcement [6].

Moreover, there was a significant discrepancy between third‐ and fifth‐year students in correctly identifying the emergency activation number (“2222”). Although fifth‐year students performed better, nearly half still remained uncertain or incorrect. This finding resonates with literature indicating that theoretical knowledge alone is insufficient without regular reinforcement and practical application [7]. Therefore, regular training and periodic refresher courses are essential to maintain adequate preparedness and proficiency in emergency situations.

Regarding specific responsibilities during emergencies, students largely recognised their initial role in providing immediate interventions, such as initiating chest compressions and effectively communicating patient information to the arriving emergency team. However, opinions were notably divided regarding active assistance during team interventions. Furthermore, most students did not recognise patient transfer to intensive care as their responsibility. Literature from various international contexts also suggests similar uncertainties among dental professionals regarding their roles during medical emergencies, advocating for clear, institution‐specific guidelines and regular, hands‐on training exercises to clarify roles and responsibilities [6, 7, 8].

## 5 Strengths and Limitations

A major strength of this study is the inclusion of both preclinical and clinical students, enabling direct comparison of awareness levels by educational stage. However, the study was limited by its single‐center design and relatively small sample size, which may affect the generalizability of the findings. Additionally, the study assessed only self‐reported knowledge without evaluating practical competency through simulations or clinical scenarios.

An additional limitation is that faculty members' knowledge and awareness of institutional emergency codes were not evaluated in this study. Given that faculty serve as primary role models and supervisors during clinical training, their level of preparedness may directly influence students' exposure and learning. Future studies should consider assessing faculty knowledge to ensure alignment between instructional expectations and clinical supervision practices.

## 6 Recommendations

Future research should include multi‐center studies involving larger and more diverse populations. Additionally, evaluating students' practical performance in simulated Code Blue scenarios would provide more comprehensive insights into their actual preparedness. Curriculum revisions incorporating structured emergency response training at earlier stages in dental education may enhance patient safety and improve outcomes in clinical settings.

Based on the findings of this pilot project, discussions have been initiated within the faculty regarding potential curriculum enhancements. Proposed revisions include the earlier introduction of institutional emergency code training during the preclinical years, incorporation of structured simulation‐based Code Blue scenarios, and periodic reinforcement sessions throughout clinical training. The goal is to ensure that all students acquire not only theoretical awareness but also practical readiness in managing in‐hospital emergencies. Future implementation of these modifications will require collaboration between dental educators, anesthesiology teams, and institutional safety committees.

In addition to early didactic integration, restructuring of the clinical curriculum is also being considered. Planned modifications include the incorporation of mandatory mock Code Blue drills during clinical rotations, structured interprofessional simulation sessions in collaboration with anesthesiology teams, and formative assessments evaluating students' response accuracy and decision‐making in simulated emergency scenarios. Embedding emergency code activation within clinical competency evaluations may further reinforce accountability and practical application. These steps aim to move beyond passive awareness toward active clinical readiness.

While recognition of Code Blue activation criteria represents an essential first step, effective emergency response also requires immediate management competence. Based on the present findings, plans are being considered to strengthen hands‐on emergency training through high‐fidelity simulation sessions, structured CPR skill assessments, and scenario‐based evaluations focusing on airway management, AED use, and team communication. Integrating objective skill‐based assessments into clinical competency requirements may further ensure that students are not only aware of emergency codes but are also capable of initiating appropriate immediate interventions.

## 7 Conclusion

In conclusion, although senior dental students demonstrated significantly better emergency code awareness and knowledge compared to junior students, considerable gaps remain. These gaps emphasise the necessity for structured, continuous, and scenario‐based educational interventions throughout dental training programs. Enhanced training involving regular refreshers and practical simulations could significantly improve dental students' preparedness, competence, and confidence in effectively responding to clinical emergencies.

## Funding

The authors have nothing to report.

## Conflicts of Interest

The authors declare no conflicts of interest.

## Data Availability

The data that support the findings of this study are available from the corresponding author upon reasonable request.
